# EpCAM-independent isolation of circulating tumor cells with epithelial-to-mesenchymal transition and cancer stem cell phenotypes using ApoStream^®^ in patients with breast cancer treated with primary systemic therapy

**DOI:** 10.1371/journal.pone.0229903

**Published:** 2020-03-26

**Authors:** Fanny Le Du, Takeo Fujii, Kumiko Kida, Darren W. Davis, Minjeong Park, Diane D. Liu, Weiguo Wu, Mariana Chavez-MacGregor, Carlos H. Barcenas, Vicente Valero, Debu Tripathy, James M. Reuben, Naoto T. Ueno

**Affiliations:** 1 Department of Breast Medical Oncology, Section of Translational Breast Cancer Research, The University of Texas MD Anderson Cancer Center, Houston, Texas, United States of America; 2 Department of Medical Oncology, Eugène Marquis Cancer Center, Rennes, France; 3 ApoCell, Inc., Houston, Texas, United States of America; 4 Department of Biostatistics, The University of Texas MD Anderson Cancer Center, Houston, Texas, United States of America; 5 Department of Hematopathology, The University of Texas MD Anderson Cancer Center, Houston, Texas, United States of America; American Society for Investigative Pathology, UNITED STATES

## Abstract

**Background:**

Tumor cells with a mesenchymal phenotype and/or cancer stem-like cells (CSCs) are known to contribute to metastasis and drug resistance. Circulating tumor cells (CTCs) undergoing epithelial-mesenchymal transition (EMT) and CTCs reflecting a dedifferentiated CSC phenotype may not be detected using only an anti-EpCAM antibody to capture them. We used an antibody-independent CTC enrichment platform, ApoStream^®^, which does not rely on any antibody, including anti-EpCAM, to capture EMT- and CSC-CTCs in breast cancer patients who received neoadjuvant chemotherapy and correlated them to pathological complete response (pCR).

**Methods:**

Blood samples from newly diagnosed breast cancer patients were prospectively collected before neoadjuvant chemotherapy (T_0_), after chemotherapy but before surgery (T_1_), and after surgery (T_2_) and processed using ApoStream. CTCs detected were stained with additional markers to define 3 CTC subsets with the following phenotypes: epithelial CTCs (CK+, EpCAM+ or E-cadherin+), EMT-CTCs (β-catenin+ or vimentin+), and CSC-CTCs (CD44+ and CD24^low^).

**Results:**

We enrolled 55 patients, 47 of which had data for analysis. EMT-CTCs were detected in 57%, 62%, and 72% and CSC-CTCs in 9%, 22%, and 19% at the T_0_, T_1_, and T_2_ time points, respectively. Counts of epithelial (P = 0.225) and EMT (P = 0.522) phenotypes of CTCs at T_0_ did not significantly predict pCR. Moreover, no correlation between CTC count change and pCR was demonstrated.

**Conclusions:**

ApoStream was successful in detecting EMT-CTCs among patients after neoadjuvant chemotherapy. However, EMT-/CSC-CTC counts did not correlate with pCR. Due to the small sample size and heterogeneity of this patient population, further study in a larger cohort of molecularly homogeneous patients is warranted.

## Introduction

Circulating tumor cells (CTCs) are found in human blood; thus, their detection might be used as a marker of early relapse [1,2]. The presence of CTCs prior to and after systemic therapy has also been reported to be a surrogate marker for poor prognosis in early breast cancer and has been linked to shorter survival in patients with metastatic breast, prostate, lung, and colorectal cancers, indicating that CTC detection could be a tool for early assessment of treatment efficacy [1–8]. While CTCs seem to provide prognostic information, their clinical utility in routine practice is yet to be established, and CTCs are not routinely used. Indeed, data from the phase III SWOG0500 trial show that a change in the chemotherapy regimen based on CTC elevation did not improve overall survival of patients with metastatic breast cancer [9]. Results of the CirCe01 phase III study show that early changes in CTC counts during third-line chemotherapy were correlated with treatment outcome [10].

So far, the only U.S. Food and Drug Administration (FDA)-approved platform for CTC detection is the CellSearch system, which targets expression of the cell-surface epithelial cell adhesion molecule (EpCAM) for CTC enrichment. However, this technique may not capture other CTC subsets in which epithelial markers are downregulated. Cells that have undergone epithelial-to-mesenchymal transition (EMT) are known to be highly aggressive and contribute to metastasis [11–13]. Moreover, the process of EMT can generate cells with stem cell-like properties, cancer stem cells (CSCs) [14], known to play a role in the metastatic process by promoting proliferation and differentiation [15]. CSC-CTCs have been detected in both primary and metastatic breast cancers [16,17]. In the metastatic setting, detection of CSC-CTCs and EMT-CTCs was associated with resistance to chemotherapy; CTCs with CSC and EMT markers were found in the blood of 74% of patients who did not respond to chemotherapy and 10% of patients who responded [16,18]. However, data on the predictive value of EMT-CTCs and CSC-CTCs in the early disease setting are not available.

Based on these reports, we hypothesized that EMT-CTCs and CSC-CTCs could predict response to neoadjuvant chemotherapy in breast cancer. To test our hypothesis, our objective in the current study was to correlate EMT-CTC and CSC-CTC counts in patients with primary breast cancer who achieved a pathological complete response (pCR) with neoadjuvant systemic treatment.

## Materials and methods

### Patients

The Institutional Review Board of The University of Texas MD Anderson Cancer Center approved this prospective study (PA12-0453). We enrolled patients with newly diagnosed histologically confirmed primary invasive breast cancer who were scheduled to undergo neoadjuvant systemic therapy followed by definitive surgery at The University of Texas MD Anderson Cancer Center. All patients, recruited by a medical oncologist in the Department of Breast Medical Oncology, signed a written informed consent before providing blood. We collected age, clinical stage, estrogen receptor (ER) and progesterone receptor (PR) status, HER2 status, Ki67 proliferation index, and Nottingham grade index (NGI), type and date of surgery, and neoadjuvant and adjuvant systemic and local treatments received from the patients’ medical records. Hormone receptor (HR) positivity was defined as ≥10% of cells having positive immunohistochemical (IHC) staining for ER and/or PR. HER2 positivity was defined as a HER2/CEP17 fluorescence in situ hybridization (FISH) ratio of ≥2.0 and/or an IHC staining score of 3+.

### Blood collection and sample processing

Three 8-ml CPT tubes of blood from newly diagnosed breast cancer patients were collected before neoadjuvant systemic treatment (T_0_); two CPT tubes were collected after completion of neoadjuvant chemotherapy and before definitive surgery (T_1_); and two CPT tubes were collected after definitive surgery and before endocrine therapy if the latter was indicated by ER status (T_2_). Blood was collected in cell-free preservative blood collection tubes. Blood samples were sent at ambient temperature to ApoCell (Houston, TX) and processed within 96 hours of collection.

### CTC enrichment using ApoStream^®^

The ApoStream^®^ platform uses a non-enrichment-based, non-biased approach using dielectrophoresis coupled with field-flow assist for the cell separation, allowing for downstream enumeration and characterization of all CTCs from the whole blood independently of EpCAM-based enrichment.

Enrichment of CTCs using the ApoStream^®^ device has been described previously [19,20]. Briefly, the Ficoll–Paque gradient separation method was used to isolate peripheral blood mononuclear cells (PBMCs). The PBMCs were suspended in ApoStream^®^ running buffer and processed on the ApoStream^®^ device. CTC-enriched isolates were collected into a microcentrifuge tube, cytospun onto a glass slide, and fixed using 2% paraformaldehyde.

### Immunofluorescent staining of CTCs and image analysis of CTC phenotypes

For CTC phenotyping and biomarker staining of ApoStream^®^-enriched CTCs, fixed cells were washed with phosphate-buffered saline (PBS), permeabilized, and blocked as described previously [19]. All antibodies were diluted in 1% Corning Human AB Serum (#45001–062, VWR, Radnor, PA)/2% normal donkey serum (#017-000-121, Jackson ImmunoResearch, West Grove, PA). After washing the cells in PBS, primary antibodies were added to each spot for immunofluorescence detection of epithelial (cytokeratin [CK], EpCAM, and E-cadherin), EMT (vimentin, β-catenin), and CSC (CD44 and CD24) phenotypes and incubated at 4°C overnight.

The ability of this platform to detect CTCs of unknown phenotypes such as CTCs with EMT or CSC features that cannot be detected based on conventional EpCAM-based enumeration has been established [21,22]. Preliminary analysis of breast cancer CTCs isolated by the ApoStream platform demonstrated that in the majority of samples, the CTCs lacked EpCAM expression [23].

### Laser scanning cytometry image analysis

An iCys laser scanning cytometer (CompuCyte, Westwood, MA) equipped with 405-nm (blue/orange emission filters), 488-nm (green/orange), and 633-nm (red) lasers and iCys 3.4.12 software was used to enumerate the three CTC phenotypes [19]. Individual CTCs were then confirmed by visual examination of each immunofluorescent antibody. The laser scanning cytometry image analysis sensitively measures protein expression levels, generating mean fluorescent intensity values that report fluorescence on a continuous scale.

Using this method, the CD45-negative cells were characterized into three CTC subsets based on protein expression levels: 1) Epithelial CTCs, defined as CK-positive [CK+] and/or EpCAM-positive [EpCAM+] and/or E-cadherin-positive [E-cadherin+] CTCs; 2) EMT-CTCs, defined as vimentin-positive [vimentin+] and/or β-catenin-positive [β-catenin+] CTCs; and 3) CSC-CTCs, defined as CD44-positive [CD44+] and CD24-low-expression [CD24^low^] CTCs. CTC positivity was defined as the detection of 1 or more CTCs for each subset.

### Study endpoints and statistical analysis

The objective of our study was to correlate EMT-CTC and CSC-CTC counts at three time points with response to neoadjuvant systemic therapy in patients with primary breast cancer. Summary statistics such as mean, median, range, frequency, and percentage were provided to describe the CTC counts (total CTCs and subsets of epithelial, EMT-, and CSC-CTCs) and patients’ demographic and clinical characteristics, such as age, gender, race, menopausal status, histology, clinical stage, clinical T classification, clinical N classification, ER status, HER2 status, NGI, Ki67 proliferation index, neoadjuvant systemic treatment, pCR status, adjuvant radiotherapy, adjuvant chemotherapy, adjuvant anti-HER2 targeted therapy, and adjuvant endocrine therapy. Wilcoxon rank sum test was used to compare continuous variables between patients with pCR vs patients with residual disease. Univariate exact logistic regression analysis was used to explore the association between the demographic / clinical characteristics and pathological complete response (pCR). Repeated measures analysis was used to evaluate changes in CTCs over time by pCR status. P<0.05 was considered statistically significant. All computations were carried out in SAS 9.4 (SAS Institute Inc., Cary, NC, USA).

## Results

### Patient demographics

A total of 55 patients were enrolled after providing written informed consent from August 2013 to May 2015. Of these 55 patients, 6 patients did not have surgery (5 were found to have metastatic disease and 1 did not return for treatment at our institution) and were excluded from the analysis. Of the 49 eligible patients, blood samples were collected from 47 patients before chemotherapy (T_0_), 37 patients after chemotherapy and before surgery (T_1_), and 31 patients after surgery (T_2_).

Patient characteristics are summarized in Table 1. The median age was 49 years (range 29–79). Thirteen patients (27%) had clinical stage II, 34 patients (70%) had stage III, and 2 (4%) had stage IV breast cancer but had local surgery performed due to oligometastatic disease. These 2 patients with stage IV disease were not excluded in our analysis in order that our cohort be considered representative of the larger population. Fifteen patients (30%) had ER-positive HER2-negative breast cancer, 17 (35%) had HER2-positive disease, of whom 5 were also ER-positive, and 17 (35%) had triple-negative breast cancer (TNBC). All patients received chemotherapy as neoadjuvant systemic treatment except for 1 patient who received neoadjuvant endocrine therapy. All patients with HER2-positive breast cancer received at least one anti-HER2 targeted therapy in the neoadjuvant setting. Of patients whose chemotherapy regimens were known, 98% received a taxane-based regimen, and 89% received anthracyclines.

**Table 1:** Baseline demographic and clinical characteristics of all eligible patients.

Characteristic	No. (%) (n = 49)
**Age, years**	
Median	49
Range	(29–79)
**Histology**	
Invasive ductal carcinoma	34 (69%)
Invasive lobular carcinoma	2 (4%)
Invasive mixed lobular and ductal carcinoma	1 (2%)
Histological inflammatory breast cancer	12 (25%)
**Clinical stage**	
II	13 (27%)
III	34 (70%)
IV	2 (4%)
**Clinical T classification**	
** T1**	1 (2%)
** T2**	15 (31%)
T3	4 (8%)
T4	1 (2%)
T4d	28 (57%)
**Clinical N classification**	
** N0**	7 (14%)
** N1**	17 (35%)
N2	6 (12%)
N3	19 (39%)
**ER ≥10%**	
Positive	20 (41%)
Negative	29 (59%)
**HER2 (IHC+ or FISH+)**	
Positive	17 (35%)
Negative	32 (65%)
**Subtypes**	
HR+	15 (30%)
HER2+	17 (35%)
TNBC	17 (35%)
**Proliferation index (Ki67 expression)**	
Median	55
Range	(10–99)
**Nottingham Grade Index (NGI)**	
I	1
II	12
III	33
Missing data	3
**Neoadjuvant systemic treatment**	
Endocrine therapy	1 (2%)
T alone	1 (2%)
A + T	14 (29%)
A + T + Carboplatin	14 (29%)
A + T + Trastuzumab	3 (6%)
A + T + Dual anti-HER2 treatment	10 (20%)
T + Dual anti-HER2 treatment	3 (6%)
T + Carboplatin + Dual anti-HER2 treatment	1 (2%)
A + Eribulin	1 (2%)
Missing data	1 (2%)
**pCR status**	
No pCR	33 (70%)
pCR	14 (30%)
Missing data	2

ER, estrogen receptor; IHC, immunohistochemistry; FISH, fluorescence in situ hybridization; HR, hormone receptor; A, anthracycline; T, taxane; pCR, pathological complete response

In the adjuvant setting, only 1 patient received additional chemotherapy; 42 patients (89%) had radiation therapy. Of the 20 ER-positive patients, 18 had endocrine therapy. Among HER2-positive breast cancer patients, only 12 out of 17 had adjuvant anti-HER2 targeted therapy.

### CTC detection

**Fig 1:**
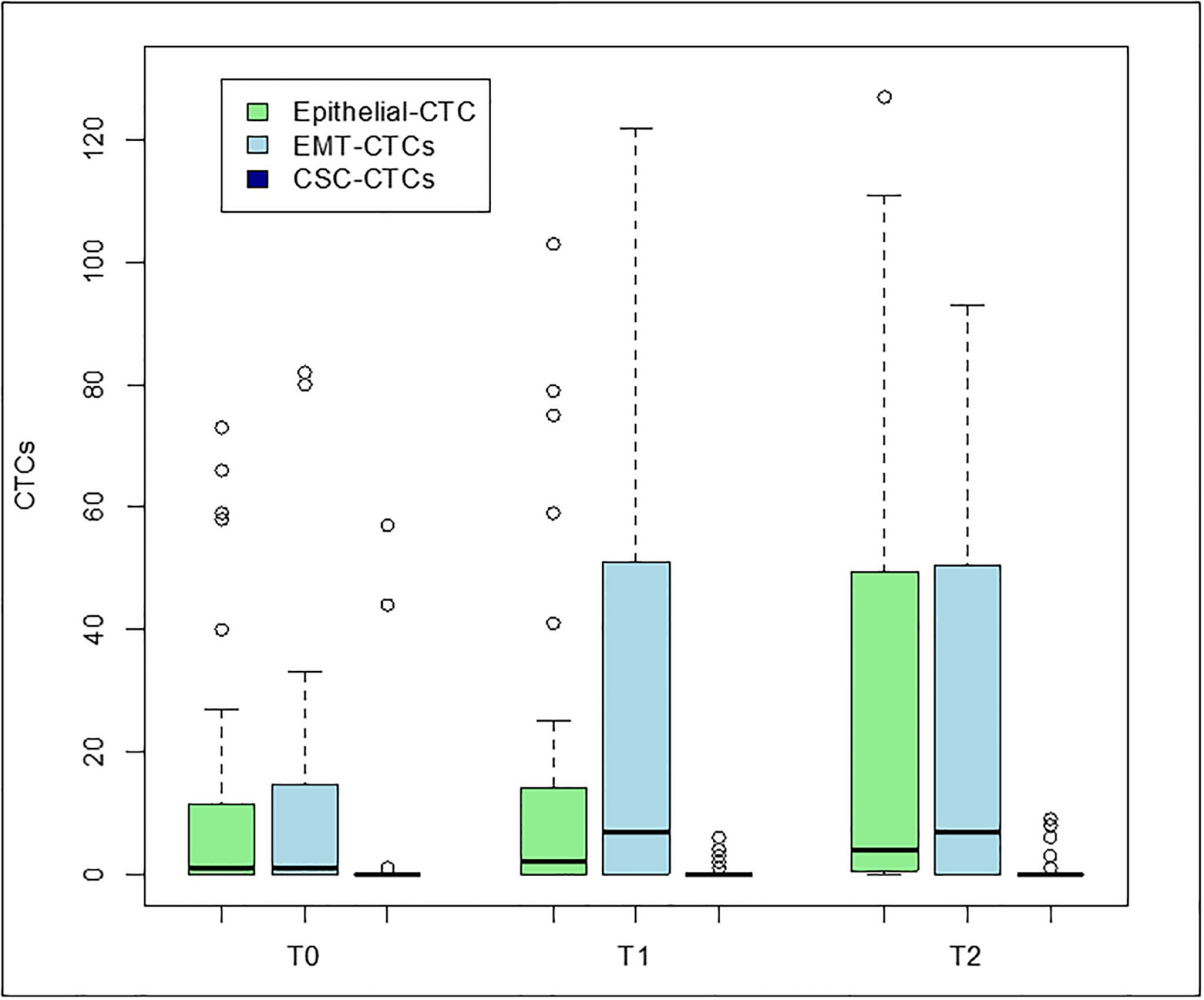
Epithelial CTC, EMT-CTC, and CSC-CTC levels at baseline (T_0_), after chemotherapy (T_1_), and after surgery (T_2_). The bottom and the top of each box represent the 25th and 75th percentiles, and the horizontal band within the box represents the median. The upper whisker represents the 75th percentile plus 1.5 times the interquartile range. The lower whisker represents the 25th percentile minus 1.5 times the interquartile range.

The detection rates of any type of CTCs increased during treatment, with a detection rate of 66% (31 of 47 samples) at T_0_, 78% (29 of 37 samples) at T_1_, and 84% (26 of 31 samples) at T_2_ (S1 Table). Epithelial CTCs were detected in 55% (26 of 47 samples), 65% (24 of 37 samples), and 74% (23 of 31 samples) of the T_0_, T_1_, and T_2_ samples, respectively. EMT-CTCs were detected in 57%, 62%, and 72% of these samples, respectively. All patients who had at least one CTC had epithelial and/or EMT-CTCs; no patient had only CSC-CTCs. The detection rates of CSC-CTCs were 9% (4 of 47 samples), 22% (8 of 37 samples), and 19% (6 of 31 samples) at time points T_0_, T_1_, and T_2_, respectively. Fig 1 shows levels of CTCs by phenotype at each time point.

We also looked at detection rate in the IBC subpopulation and in the different breast cancer subtypes. The CTC detection rates in IBC patients were similar to those in the overall population (S2 Table). In the TNBC subgroup, detection of CTCs at baseline trended to be lower than in other subgroups (44% of the samples versus 66% and 88% in the ER-positive and HER2-positive subgroups, respectively; S3 Table). On the contrary, after surgery, detection of EMT-CTCs was more frequent in TNBC (90% of the samples vs 50% and 55% in the ER-positive and HER2-positive subgroups, respectively).

### CTCs, prognostic factors, and pCR

When we looked at the relationship between key clinical prognostic factors and CTCs, we found that EMT-CTCs at T_0_ (P = 0.022) were more likely to be detected at higher clinical stage. However, no other significant interactions were detected.

Neither epithelial (P = 0.225) nor EMT (P = 0.522) phenotypes of CTC counts, at T_0_, were significantly different between pCR and non-pCR groups.

We also used exact logistic regression analysis to estimate odds ratios, due to small numbers. We did not find any significant association between baseline epithelial CTCs or EMT-CTCs and pCR. Two variables, ER and PR positivity, were significantly associated with pCR. Patients were 0.077 times less likely to achieve a pCR if they had ER-positive tumors than if they had ER-negative tumors (p = 0.0091). Patients were 0.087 times less likely to achieve a pCR if they had PR-positive tumors than if they had PR-negative tumors (p = 0.0095) (Table 2).

**Table 2:** Univariate logistic regression analysis on pCR (Y [pCR] vs N [no pCR]).

Variables	Levels	Odds Ratio (95% CI)	P-value
Age (N = 45)		0.967 (0.91–1.023)	0.2555
Menopausal status (N = 44)	Post vs Peri	0.412 (0–7.824)	0.5833
	Pre vs Peri	0.5 (0–9.5)	0.6667
ER expression (%) (N = 45)		0.949 (0.896–1.005)	0.0727
ER positivity (N = 45)	Pos vs Neg	0.077 (0.002–0.637)	***0*.*0091***
PR expression (%) (N = 45)		0.788 (0.554–1.122)	0.1868
PR positivity (N = 45)	Pos vs Neg	0.087 (0–0.462)	***0*.*0095***
HER2 (N = 45)	Pos vs Neg	1.365 (0.277–6.291)	0.8945
Ki67 (N = 45)		1.014 (0.977–1.052)	0.4761
Clinical T classification (N = 45)	T3 or T4 vs T1 or T2	1.174 (0.250, 6.439)	1.000
Clinical N classification (N = 45)	N1 vs N0	0.739 (0.067–11.208)	1.0000
	N2 vs N0	1.299 (0.059–29.113)	1.0000
	N3 vs N0	0.724 (0.072–10.364)	1.0000
Grade (N = 42)	>2 vs ≤2	7.056 (0.820, 341.457)	0.0907
Surgery (N = 45)	BCS vs Mastectomy	0.457 (0.009–4.767)	0.8653
Epithelial CTCs at T_0_ (N = 43)		0.989 (0.964–1.016)	0.4350
EMT-CTCs at T_0_ (N = 43)		0.977 (0.936–1.020)	0.2874

ER, estrogen receptor; PR, progesterone receptor; BCS, breast conserving surgery; CTC, circulating tumor cell; EMT, epithelial-mesenchymal transition

### Longitudinal CTC counts

**Fig 2:**
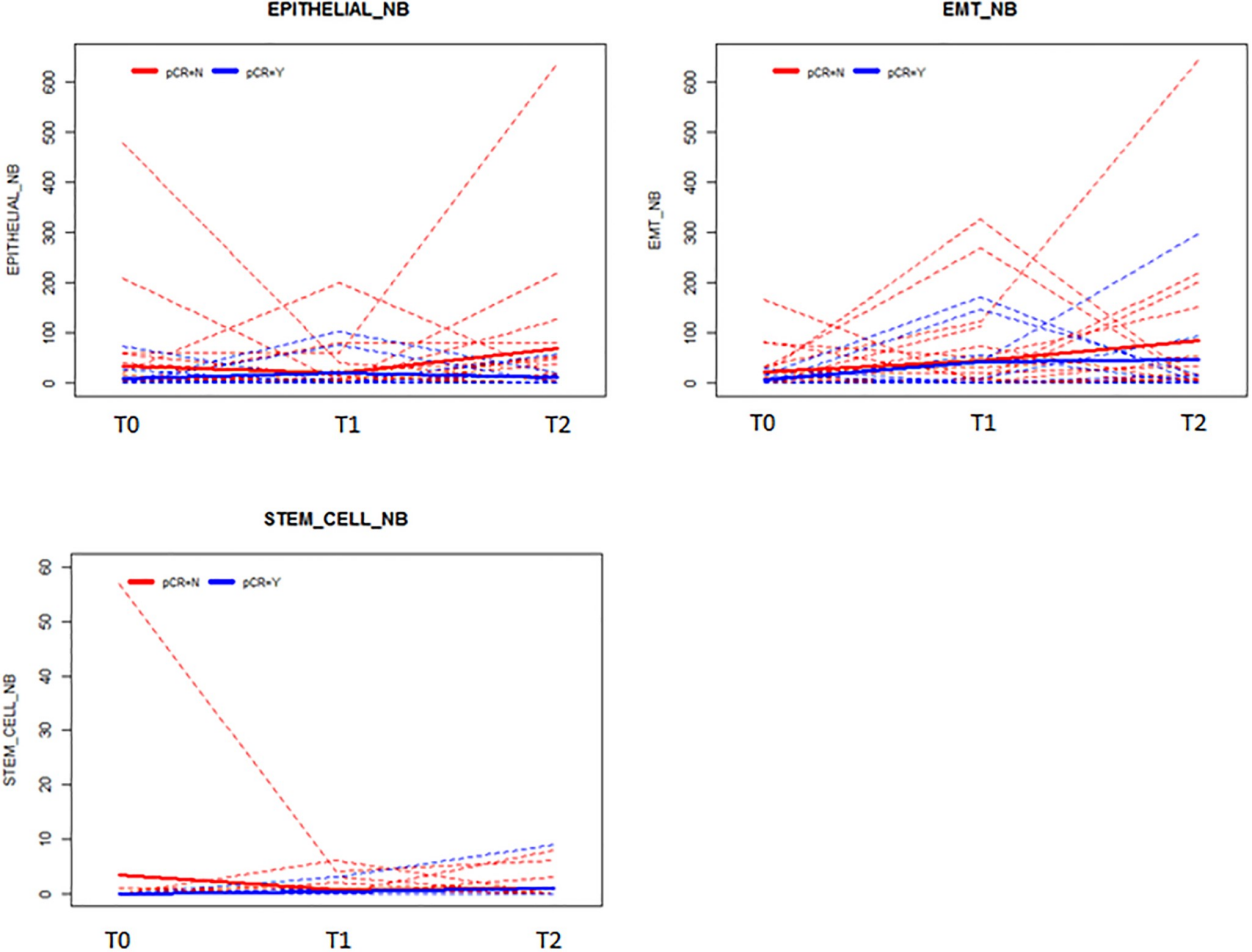
CTC counts over time by pCR status. Shown are epithelial CTC (a), EMT-CTC (b), and CSC-CTC (c) counts at 3 times points: baseline (T_0_), after chemotherapy (T_1_), and after surgery (T_2_).

One of the aims of our study was to look at CTC counts over time. Fig 2 highlights the evolution of epithelial CTCs, EMT-CTCs, and CSC-CTCs over time for each patient, by pCR status. No statistically significant associations between CTC counts over time and pCR were demonstrated by repeated measures analysis.

## Discussion

In this study, we applied the ApoStream platform, which uses a non-enrichment-based non-biased approach, to identify CTCs in patients with not only advanced but also early-stage breast cancer and were able to identify CTCs that had undergone EMT as well as those that had acquired a CSC phenotype. We detected traditional epithelial CTCs in 55 to 74% of the 47 patients analyzed in this study, depending on the time of the sample collection. We also detected EMT-CTCs in 57 to 72% of these patients. CSC-CTCs were detected in 9 to 22% of the patients. The CSC-CTC detection rate in our study was low compared to the 51% detection rate reported with the AdnaTest EMT2 kit, which employs a cocktail of antibodies (anti-EpCAM, anti-EGFR, and anti-HER2) to capture CTCs and transcripts of ALDH1 as a CSC marker [24]. The increasing number of patients with detectable CTCs through neoadjuvant therapy is concordant with previous data of our group showing that neoadjuvant therapy was unable to eliminate CTCs undergoing EMT [25]. Such data are not surprising given that in breast cancer, the EMT state has been associated with CSC properties, including self-renewal capabilities and resistance to conventional therapies [26].

However, we were not able to demonstrate that epithelial CTCs, EMT-CTCs, or CSC-CTCs serve as surrogate markers for pCR. In agreement, ancillary studies of the phase III GeparQuattro and Neo ALTTO trials were not able to confirm an association either; a decrease in the CTC count after neoadjuvant treatment in locally advanced breast cancer was not significantly associated with better response to systemic treatment [27–29]. In a more recent meta-analysis of 2090 patients treated with neoadjuvant chemotherapy, CTC detection—before neoadjuvant chemotherapy or before surgery—by CellSearch was also not able to demonstrate a correlation between CTC numbers and pCR [30]. One explanation could be the increased numbers of CTCs detected during treatment in our study, whereas most of the studies highlighted a decrease in CTCs during neoadjuvant treatment that reflected tumor load [28,30,31]. This higher CTC detection rate over treatment could be due to the high vascularity and metastatic potential of inflammatory breast cancer (IBC)[27], which accounted for more than half of our enrolled patients. A similar increase in CTCs during treatment was demonstrated in the ancillary study of the Neo ALTTO trial [27]. Another explanation could be our small sample size, which was not powered to correlate specific subtypes of CTCs with specific breast cancer subtypes, and, also, our inability to detect relevant CTC subtypes. CTCs are known to be very heterogeneous, and our ongoing effort is aimed at validating the definition of phenotypes used to characterize circulating cancer cells at various stages of disease. Further studies are warranted to investigate the use and characterization of additional antibodies that may be used to define relevant CTC subtypes that were not included in this specific analysis.

The IMENEO meta-analysis was able to demonstrate a significant impact of CTC detection with regard to overall survival and distant disease–free survival [30]. We were not able to perform such an analysis due to the lack of events to allow conclusions on disease-free or overall survival. Mature data are warranted to assess the predictive value of EMT- or CSC-CTCs in the overall population [30].

In our cohort, we had a large number of clinical IBCs (28 of 49 patients), a factor that needs to be considered when interpreting our findings, even though, in our exploratory subgroup analysis, enumeration of CTCs for IBC patients did not seem to differ from that of the overall population, contrary to what we could expect from the literature [30]. However, our study was not powered to detect such a difference in this specific population. CTCs are known to be detected in a large number of patients with newly diagnosed metastatic IBC (84%, versus 55% of stage III patients) [32]. In a pooled analysis of two prospective trials in patients with non-metastatic IBC, using bevacizumab, the CTC detection rate proved to be an independent prognostic factor of overall survival [31]. Moreover, association of pCR status and CTC detection at baseline helped isolate a subgroup of IBC patients with excellent survival (94% 3-year overall survival) [31]. However, using CTCs to predict response remains a challenge in the clinical setting.

The analytic and clinical validity of the CTC assay are now established; however, the clinical utility of CTCs has not been demonstrated. Only one study specifically designed to investigate the role of CTCs in decision making has been published, and the results were negative. In that study of patients with metastatic breast cancer, switching the chemotherapy regimens of patients with persistently high CTC counts did not improve overall survival [9,33].

However, technology now exists to characterize CTCs rather than simply count them [33,34]. Implementation of biomarkers in CTCs related to drug mechanism or pathway resistance may be useful for assessing clinical response. In a large phase III breast cancer trial (BEACON) with 800 patients, ApoStream^®^ was used to isolate CTCs. These cells were further characterized by measuring DNA damage biomarkers. A strong correlation was observed in ApoStream^®^-isolated CTCs that were positive for topoisomerase I and overall survival in a subset of patients treated with the investigational drug (pegylated form of irinotecan) [19].

## Conclusion

Our study suggests that we have the ability to detect, in the neoadjuvant setting, chemoresistant micrometastatic disease expressing an EMT-like or CSC-like phenotype. However, neither EMT-CTCs nor CSC-CTCs were able to predict tumor response to neoadjuvant chemotherapy. Because of the heterogeneity of this patient population and small sample size, further studies are needed in a larger patient cohort with molecularly homogeneous patients.

## Supporting information

S1 Table Detection rate (≥1 cell) and mean number (range) of CTCs detected for each CTC phenotype among the full study cohort (47 patients). (DOCX)

S2 Table Detection rate (≥1 cell) and mean number (range) of CTCs detected for each CTC phenotypes among the IBC population (28 patients). (DOCX)

S3 Table Detection rate (≥1 cell) and mean number (range) of CTCs detected for each CTC phenotype by breast cancer subtype. (DOCX)

S1 Data (XLS)
