# Supplementary material for: EpCAM-independent isolation of circulating tumor cells with epithelial-to-mesenchymal transition and cancer stem cell phenotypes using ApoStream® in patients with breast cancer treated with primary systemic therapy
Source: PLoS One. 2020 Mar 26;15(3):e0229903. doi: 10.1371/journal.pone.0229903 (PMC7098555; doi:10.1371/journal.pone.0229903)
Supplement: S1 Table — (DOCX) [file pone.0229903.s001.docx]

**S1 Table -** Detection rate (≥1 cell) and mean number (range) of CTCs detected for each CTC phenotype among the full study cohort.

| **Time point** | **CTC phenotype** | | | |
| --- | --- | --- | --- | --- |
|  | **All CTCs** | **Epithelial** | **EMT** | **CSC** |
| **T_0_**  **No of patients (%)**  Mean (range) N=47 | **31 (66%)** | **26 (55%)**  24 (0-477) | **27 (57%)**  16 (0-167) | **4 (9%)**  2 (0-57) |
| **T_1_**  **No of patients (%)**  Mean (range) N=37 | **29 (78%)** | **24 (65%)**  19 (0-201) | **23 (62%)**  49 (0-327) | **8 (22%)**  0.6 (0-6) |
| **T_2_**  **No of patients (%)**  Mean (range) N=31 | **26 (84%)** | **23 (74%)**  49 (0-637) | **21 (72%)**  72 (0-645) | **6 (19%)**  0.9 (0-9) |
