# Supplementary material for: EpCAM-independent isolation of circulating tumor cells with epithelial-to-mesenchymal transition and cancer stem cell phenotypes using ApoStream® in patients with breast cancer treated with primary systemic therapy
Source: PLoS One. 2020 Mar 26;15(3):e0229903. doi: 10.1371/journal.pone.0229903 (PMC7098555; doi:10.1371/journal.pone.0229903)
Supplement: S2 Table — (DOCX) [file pone.0229903.s002.docx]

**S2 Table -** Detection rate (≥1 cell) and mean number (range) of CTCs detected for each CTC phenotype among the IBC population (28 patients).

| **Time point** | **CTC phenotype** | | | |
| --- | --- | --- | --- | --- |
|  | **All CTCs** | **Epithelial** | **EMT** | **CSC** |
| **T_0_**  **No of patients (%)**  Mean (range) N=26 | **18 (69%)** | **14 (54%)**  19 (0-208) | **16 (62%)**  21 (0-167) | **3 (12%)**  2 (0-44) |
| **T_1_**  **No of patients (%)**  Mean (range) N=25 | **20 (80%)** | **15 (60%)**  13 (0-103) | **16 (64%)**  49 (0-269) | **5 (20%)**  0.5 (0-6) |
| **T_2_**  **No of patients (%)**  Mean (range) N=16 | **12 (75%)** | **11 (69%)**  74 (0-637) | **11 (69%)**  42 (0-297) | **1 (6%)**  0.6 (0-9) |
