# Supplementary material for: EpCAM-independent isolation of circulating tumor cells with epithelial-to-mesenchymal transition and cancer stem cell phenotypes using ApoStream® in patients with breast cancer treated with primary systemic therapy
Source: PLoS One. 2020 Mar 26;15(3):e0229903. doi: 10.1371/journal.pone.0229903 (PMC7098555; doi:10.1371/journal.pone.0229903)
Supplement: S3 Table — (DOCX) [file pone.0229903.s003.docx]

**S3 Table -** Detection rate (≥1 cell) and mean number (range) of CTCs detected for each CTC phenotype by breast cancer subtype.

| **Time point** | **CTC phenotype** | | | |
| --- | --- | --- | --- | --- |
|  | **All CTCs** | **Epithelial** | **EMT** | **CSC** |
| **T_0_** | | | | |
| **ER+**  N=15 | 10 (66%) | 10 (66%) | 8 (53%) | 1 (7%) |
| **HER2+**  N=16 | 14 (88%) | 11 (69%) | 12 (75%) | 2 (13%) |
| **TNBC** N=16 | 7 (44%) | 5 (31%) | 6 (38%) | 1 (6%) |
| **T_1_** | | | | |
| **ER+**  N=11 | 8 (73%) | 6 (55%) | 7 (64%) | 1 (9%) |
| **HER2+**  N=15 | 13 (87%) | 11 (74%) | 9 (60%) | 4 (27%) |
| **TNBC** N=11 | 8 (73%) | 7 (64%) | 7 (64%) | 3 (27%) |
| **T_2_** | | | | |
| **ER+**  N=10 | 7 (70%) | 7 (70%) | 5 (50%) | 2 (20%) |
| **HER2+**  N=9 | 8 (89%) | 7 (78%) | 5 (55%) | 1 (11%) |
| **TNBC** N=10 | 9 (90%) | 8 (80%) | 9 (90%) | 2 (20%) |
